# NIEHS/FDA CLARITY-BPA research program update

**DOI:** 10.1016/j.reprotox.2015.07.075

**Published:** 2015-07-29

**Authors:** Jerrold J. Heindel, Retha R. Newbold, John R. Bucher, Luísa Camacho, K. Barry Delclos, Sherry M. Lewis, Michelle Vanlandingham, Mona I. Churchwell, Nathan C. Twaddle, Michelle McLellen, Mani Chidambaram, Matthew Bryant, Kellie Woodling, Gonçalo Gamboa da Costa, Sherry A. Ferguson, Jodi Flaws, Paul C. Howard, Nigel J. Walker, R. Thomas Zoeller, Jennifer Fostel, Carolyn Favaro, Thaddeus T. Schug

**Affiliations:** a National Institute of Environmental Health Sciences/National Institutes of Health, Division of Extramural Research and Training, Research Triangle Park, NC 27709, United States; b National Institute of Environmental Health Sciences/National Institutes of Health, Division of the National Toxicology Program, Research Triangle Park, NC 27709, United States; c Division of Biochemical Toxicology, National Center for Toxicological Research, U.S. Food and Drug Administration, Jefferson, AR 72079, United States; d Division of Neurotoxicology, National Center for Toxicological Research, U.S. Food and Drug Administration, Jefferson, AR 72079, United States; e Department of Comparative Biosciences, University of Illinois, Urbana, IL 61802, United States; f Office of Scientific Coordination, National Center for Toxicological Research, U.S. Food and Drug Administration, Jefferson, AR 72079, United States; g Biology Department, University of Massachusetts, Amherst, MA 01003, United States; h Team Vistronix, NTP Computer and User Support, National Institute of Environmental Health Sciences/National Institutes of Health, Division of the National Toxicology Program, Research Triangle Park, NC 27709, United States

**Keywords:** Bisphenol A, NIEHS, FDA, NTP, CLARITY-BPA, Consortium, Endocrine disruptors

## Abstract

Bisphenol A (BPA) is a chemical used in the production of numerous consumer products resulting in potential daily human exposure to this chemical. The FDA previously evaluated the body of BPA toxicology data and determined that BPA is safe at current exposure levels. Although consistent with the assessment of some other regulatory agencies around the world, this determination of BPA safety continues to be debated in scientific and popular publications, resulting in conflicting messages to the public. Thus, the National Toxicology Program (NTP), National Institute of Environmental Health Sciences (NIEHS), and U.S Food and Drug Administration (FDA) developed a consortium-based research program to link more effectively a variety of hypothesis-based research investigations and guideline-compliant safety testing with BPA. This collaboration is known as the Consortium Linking Academic and Regulatory Insights on BPA Toxicity (CLARITY-BPA). This paper provides a detailed description of the conduct of the study and a midterm update on progress of the CLARITY-BPA research program.

## 1. Introduction

Data available for the assessment of the safety of chemicals often includes results from regulatory guideline-compliant studies performed or sponsored by government agencies or commercial entities involved in the production or use of chemicals, and other studies characterized as hypothesis-driven generally performed by investigators in university settings. Funding for the university-based investigators comes primarily from federal grants, although industry funding is not uncommon. The hypothesis-driven, mechanistically focused studies are usually small in scale and often employ novel tools, up-to-date technologies, and various experimental models appropriate to study basic mechanisms, developmental effects, and physiology. In contrast, guideline-compliant studies utilize validated endpoints that are conducted to meet regulatory and statutory mandates. Guideline-compliant studies are conducted under a rigorous set of internationally accepted procedures developed to ensure consistency and standardization in the conduct and reporting of toxicity studies for regulatory purposes. Examples of such procedures include guidelines described by the NTP [1] and the Organization for Economic Cooperation and Development [2]. Such studies are most often conducted to support pre-clinical safety screening or for hazard identification, dose-response, and safety evaluation. In addition, when conducted for regulatory purposes, the studies are conducted under Good Laboratory Practices (GLP) regulations [3] to ensure quality control and detailed record keeping. Both guideline-compliant and hypothesis-driven research make valuable contributions to our understanding of the potential adverse health effects of chemical exposures [4].

Although BPA is a well-studied chemical, there are few existing chronic toxicity data that include exposure during the perinatal period or that include doses within an order of magnitude of the level of potential human dietary exposures (estimated by the FDA to be less than 1 μg/kg bw/day, [5]). Divergent findings and interpretations of data from BPA toxicological studies exist. Indeed there is a body of literature from hypothesis-driven studies that have reported effects of low doses of BPA that constitute a hazard [6–8]. On the other hand, recent reviews of the data by regulatory agencies continue to maintain that BPA is safe at current exposure levels [1,9,10]. To address these divergent views, NIEHS and FDA agreed to perform a modified guideline-compliant chronic toxicity study, conducted under GLP regulations at an FDA facility, and involve university-based investigators who would share biological samples from that study to pursue functional, morphological, and molecular endpoints that are not typically included in guideline-compliant studies. The resulting consortium-based research program, called Consortium Linking Academic and Regulatory Insights on Toxicity of BPA (CLARITY-BPA), integrates government guideline-compliant research with 13 NIEHS-funded university-based studies. The comprehensive evaluation of guideline-compliant validated endpoints with additional endpoints in the frame of a common and robust study design is expected to significantly improve the interpretation of the wealth of data that is being generated by all consortium partners, including the characterization of the dose response of the effects observed and their interpretation in an integrated biological context. Details about the primary goals and organizational structure of the program have been previously described [11]. This report provides more in-depth details of the guideline-compliant study, the consortium logistics, and outlines the additional endpoints analyzed by those university-based researchers.

### 1.1. CLARITY-BPA program and general study design

The CLARITY-BPA program is a cooperative agreement, conducted under the auspices of the National Toxicology Program (NTP), between NIEHS-funded university-based researchers, staff at the NIEHS Division of the National Toxicology Program (DNTP) and Division of Extramural Research and Training (DERT), and staff at the FDA's National Center for Toxicological Research (NCTR). The core chronic GLP study is being performed at the NCTR and contains the essential elements of a guideline-compliant study, such as standard protocols and endpoints typically considered by regulatory agencies in hazard identification and risk assessment. The core chronic study shares many of the elements of the subchronic 90 day study that preceded it [12], several of which differ from previous guideline-compliant BPA studies, notably monitoring of BPA levels in animal housing materials and use of direct gavage dosing for postnatal exposure. This route of exposure was selected because lactational transfer of BPA has been demonstrated to be low in this model [13] and because direct ingestion of BPA leaching from food containers or packaging materials is the exposure of interest in infants. In addition, direct dosing during the neonatal period results in precise exposure levels. The core study also included limited assessments of internal dosimetry at the lowest tested doses that build upon those reported for the subchronic 90 day study [14]. In addition to the continuous daily dosing throughout the study, a stop-dose arm was included. In this stop-dose arm, dosing ended at weaning and the animals were assessed concurrently with the continuous dose animals. This design allows comparisons of the treatment effects following gestational-through-weaning exposure with those of gestation-through-lifetime exposure and thus partially addresses the impact of early exposure alone relative to lifetime exposure or adult-only continuous exposure [15]. Five dose levels of BPA, spaced at log intervals from 2.5 to 25,000 μg BPA/kg bw/day, were used to cover the wide range of doses over which BPA-induced effects have been reported in the scientific literature. Because BPA binds to the estrogen receptor and many of its reported effects have been hypothesized to be due to modulation of estrogen signaling, two dose levels of the reference estrogen ethinyl estradiol (EE_2_) were also included. The Sprague-Dawley rat from the NCTR colony was the animal model used in the current study, similar to what was done in the subchronic 90 day study [14]. This animal model has been shown to be sensitive to estrogenic compounds, including EE_2_ and genistein [16–21] and a comprehensive evaluation of the pharmacokinetics of BPA across life stages has been reported in this strain [13,14,22–24].

University-based researchers submitted grant applications in response to an NIEHS issued Funding Opportunity Announcement (RFA-ES-10-009). Grant applications were reviewed for scientific merit by a Special Emphasis Panel convened by NIEHS. FDA scientists were not involved in the selection of the projects or endpoints, except to determine the feasibility of accomplishing the goals of the projects within resource constraints (such as availability of animal room space and/or personnel). Thirteen applications were funded; some modifications to the original proposals were required to fit within the resource constraints of the overall project. The university-based researchers were provided animals and/or tissues or serum from animals that were litter mates of the core study animals; all animals were housed at NCTR and exposed to BPA under identical conditions. This integrated research plan was intended to eliminate variance in institutional and environmental conditions that potentially exist between studies conducted in different laboratories and to leverage extensive evaluations of internal dosimetry throughout the life-stages in the chosen animal model [14].

### 1.2. Dose selection

The subchronic 90 day BPA study conducted prior to CLARITY-BPA in part to select doses for the present study [12], included two negative controls (naïve or non-treated; vehicle), seven equally spaced “low” doses of BPA doses between 2.5 and 2,700 μg/kg bw/day, two “high” doses of BPA (100,000 and 300,000 μg/kg bw/day), and two doses of the reference estrogen (0.5 and 5 μg EE_2_/kg bw/day). In that study, there were clear adverse effects of BPA treatment at the two highest doses of 100,000 and 300,000 μg BPA/kg bw/day, some of which were also seen in the EE_2_ groups. Statistically significant changes at the gene expression level of siblings were detected in the “low BPA” dose range [25]. The biological meaning of these changes is unclear. Modulation of the level of estrogen receptor transcripts was further identified in the brain of female rats in this animal cohort in the “low BPA” dose range, although their neurobehavioral consequences are currently unknown [26]. Similarly, in a separate study using identical exposure conditions, statistically significant effects on estrogen receptor expression in the hypothalamus and amygdala of PND 1 pups of both sexes [27] and increased volume of the sexually dimorphic nucleus of the preoptic area in PND 21 males [28] were reported following gestational exposure to 2.5 and 25 μg BPA/kg bw/day. Based on those results and current estimates of human exposure levels [5], it was agreed by CLARITY-BPA stakeholders that logspaced doses ranging from 2.5 to 25,000 μg BPA/kg bw/day would provide the essential information sought by the study. In addition, in the subchronic 90 day study, both doses of EE_2_ showed clear effects on multiple endpoints in females, while effects on males were largely confined to the higher 5 μg EE_2_ /kg bw/day dose group. For the CLARITY-BPA study, the NIEHS-funded university-based researchers felt that a high dose of 0.5 μg EE_2_/ kg bw/day and a lower dose of 0.05 μg EE_2_/kg bw/day would be most informative regarding the sensitivity of the animal model to EE_2_. These were the two EE_2_ doses used in the CLARITY-BPA study.

## 2. Study conduct details

This section describes the study material evaluations, general study design, and animal treatments that were conducted at the NCTR to provide animals for the core chronic two year study and hypothesis-driven studies developed and performed by academic scientists.

### 2.1 Bisphenol A (BPA), ethinyl estradiol (EE_2_), and vehicle

BPA (CAS 80-05-7, TCI America Portland OR, catalog B0494, Lot 111909/AOHOK [air-milled], >99% pure) and EE_2_ (CAS 57-636, Sigma-Aldrich Chemical Co. St Louis, MO, catalog E4876, Lot 071M1492V, >99% pure) were used in these studies [12]. Purity of both compounds was verified at 6-month intervals during the study and again at the end of the core study at two years to confirm test article stability. The vehicle used to deliver BPA and EE_2_ was 0.3% aqueous carboxymethylcellulose (CMC). The CMC powder was obtained from Sigma–Aldrich (St. Louis, MO; catalogue C5013, Lot 041M0105V).

### 2.2 Diet characterization

Verified Casein Diet 10 IF, 5k96, a soy-and alfalfa-free diet provided in round pellets, γ-irradiated [Test Diets, Purina Mills, Richmond, IN; catalog 1810069; http://www.labdiet.com/cs/groups/lolweb/@labdiet/documents/web_content/mdrf/mdi4/∼edisp/ducm04_028427.pdf] was used to minimize phytoestrogen exposure. Certificates of analysis (nutrients, selected vitamins and minerals, microbiological and chemical contaminants) were provided by the manufacturer for each lot of diet prior to shipment. Extracts of each diet lot were monitored for BPA and selected myco/phytoestrogens (daidzein, genistein, coumestrol, and zearalenone) by liquid chromatography/tandem mass spectrometry (LC/MS/MS). None of the diet lots contained BPA above the protocol-specified maximum of 5 ppb [12]. Similarly, no diet lot contained phyto/mycoestrogens above the protocol-specified maximum level (2 ppm for genistein and daidzein, and 0.05 ppm for coumestrol and zearelenone).

### 2.3. Assessment of background levels of BPA in study materials other than diet

Other study materials screened for BPA included drinking water and extracts of animal bedding, polysulfone cage leachates, and the CMC dosing vehicle. In addition, the silicone water bottle stoppers were analyzed in the subchronic 90-day study [12]. Except for one batch of 0.3% CMC solution, which was excluded from the study, none of these materials had BPA levels detectable above the average analytical blank levels determined from triplicate samples on each day of analysis. Drinking water was evaluated every 3 months, and extracts of each lot of bedding were assayed; one lot of bedding had an unidentified contaminant that prevented BPA analysis and was not used. Polysulfone cages without excessive wear and scratches were used, and random samples of cage leachates were analyzed prior to the study start. BPA was not detectable in any of these analyses above the average analytical blanks.

### 2.4. Dose groups

There were 14 treatment groups in the core study and 16 treatment groups in the university-based studies, although some of the latter studies requested fewer treatment groups (see Table 1 for a detailed description of each study). Briefly, there was a vehicle control group (0.3% CMC), five BPA dose groups (2.5, 25, 250, 2,500, and 25,000 μg/kg bw/day), and two EE_2_ dose groups (0.05 and 0.5 μg/kg bw/day). Each of these eight dose groups was split into two separate groups, a continuously dosed group and a stop dose group, with the latter having treatment terminated at weaning [postnatal day (PND) 21; day of birth = PND 0]. The core chronic two year study did not include the stop dose EE_2_ arm, due to insufficient physical space, but this arm was included in the hypothesis-driven studies requiring a reference estrogen control. Additionally, one separate breeding produced vehicle controls and a BPA dose of 250,000 μg/kg bw/day for additional evaluation of testes and epididymal sperm in hypothesis driven studies.

### 2.5. Preparation and analysis of dose formulations

BPA and EE_2_ doses were prepared in the vehicle, 0.3% CMC in autoclaved Nanopure water, and administered (5 ml/kg bw) daily, seven days a week. Homogeneity and stability of the dose preparations were determined prior to the study start in a manner similar to what has been previously described [12]. Doses were prepared on an as needed basis within the stability window and the preparations were periodically certified to be ±10% of the target dose. In addition, dosing preparations were analyzed at the end of the use period for the initial batches of doses, periodically over the course of the study, and after the last dosing to verify that the correct dosing formulations were administered to the study animals.

Doses were administered by gavage with a modified Hamilton Microlab^®^ ML511C programmable 115 V pump (Hamilton Co., Reno, NV). Four separate dosing stations were used in each animal room: (1) vehicle control, (2) 2.5, 25, and 250 μg BPA/kg bw/day, (3) 2500 and 25,000 μg BPA/kg bw/day, and (4) 0.05 and 0.5 μg EE_2_/kg bw/day. Dosing was always conducted from the lowest to highest dose on any given pump, and cleaning and maintenance of the equipment were performed as described in Delclos et al. [12]. The accuracy of dose delivery from the pumps was assessed every three months and established to be within 10% of the target volume accuracy.

### 2.6. Animal source and housing conditions

All animal use and procedures for the core study were approved by the NCTR Laboratory Animal Care and Use Committee and conducted in an Association for Assessment and Accreditation of Laboratory Animal Care (AALAC)-accredited facility. Throughout the study, animal rooms were maintained at 23 ± 3 °C with a relative humidity of 50 ± 20% and food and water were available *adlibitum*. All animal rooms were under a 12 h light/dark cycle, with lights on at 6 AM, except those used to house weaned animals for the behavior study that were kept on a 12 h light/dark cycle, with lights on at 11 AM to accommodate behavioral testing during the dark phase. There was a minimum of 10 room air changes/h in all animal rooms. The source of the NCTR CD rats (Strain Code 23) used was the NCTR Rodent Breeding Colony. These breeding-source rats are routinely fed NIH-41 irradiated pellets (IRR. NIH-41, catalogue # 7919C, Harlan Laboratories, Madison, WI) and housed in polycarbonate cages with hardwood chip bedding (P.J. Murphy, Montville, NJ and Lab Animal Supplies, Inc., Lewisville, TX) and water in polycarbonate water bottles while housed in the NCTR colony.

Six hundred male and 600 female weanling (*circa* PND 21) NCTR CD rats were assigned to the study in five equal loads spaced four weeks apart (Loads 1–5). In addition, 28 females were assigned to a small separate breeding (herein referred to as “Load 0”). Once assigned to the study, rats were fed pelleted irradiated Purina 5K96 feed (Test Diets, Purina Mills, Richmond, IN), housed in polysulfone cages fitted with microisolator tops (Ancare, Corp, Bellmore, NY) with hardwood chip bedding and provided Millipore-filtered water in glass water bottles with silicone stoppers (#7721 clear, The Plasticoid Co., Elkton, MD).

Animal health was monitored throughout the core study in accordance with the Sentinel Animal Program at NCTR. Each animal room contained sentinel rats for microbiological surveillance and one sentinel animal per room was evaluated every 3 months over the course of the study. In some cases, as advised by the NCTR veterinary staff, the hardwood chip bedding of animals with external skin lesions was replaced with Alpha-Dri bedding (Shepherd Specialty Papers, Richland, MI; certified at NCTR to have BPA levels below the analytical LOD), a virgin cellulose product which was free of wood pieces that tended to embed in wounds and exacerbate inflammation. Such lesions were observed with similar frequencies in vehicle controls and all treatment groups.

For all animals, cages were changed twice weekly. Glass water bottles were changed at least once weekly to maintain a constant supply. Throughout the study, cage racks were changed every two weeks and cage locations on those racks were rotated every two weeks.

### 2.7. Animal breeding, randomized allocation to study, and dosing

**Fig. 1:**
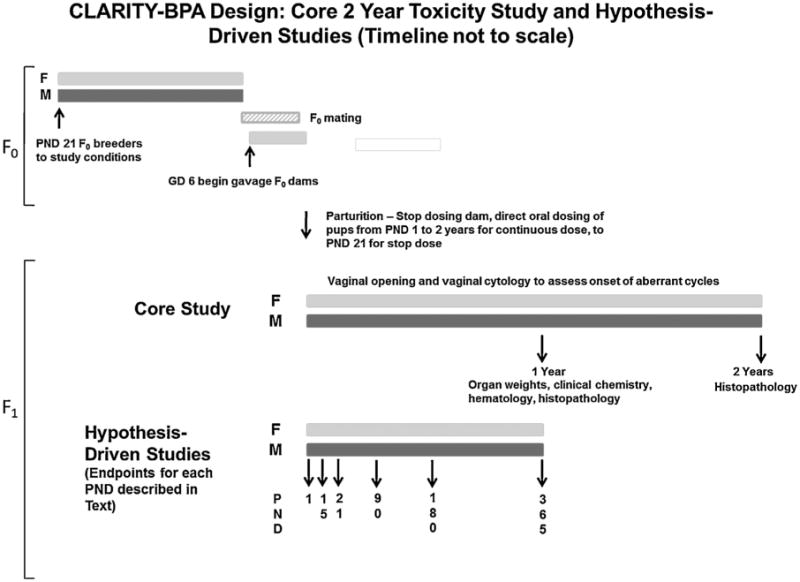
Schematic representation of the study design as described in the text. The planned animal assessment times for core chronic and specialty studies are indicated; continuous and stop dose arms are not depicted.

A summary of the experimental design is presented in Fig. 1. Approximately 2 weeks prior to mating, female breeders were randomized to treatment groups stratified by body weight to produce approximately equivalent mean body weights in each group. The pre-mating assignment of dams to dose groups was necessitated by the use of randomly cycling females and the 10 day mating period described below. Male breeders were assigned to breeding pairs with the stipulation that no sibling or first cousin mating was permitted. Rats were mated at 10–14 weeks of age for females and 11–15 weeks of age for males. As indicated above, animals were mated in five loads or cohorts spaced four weeks apart. The number of pairs assigned to treatment groups in later loads was adjusted based on the number of litters produced for the study from prior loads. Mating was conducted as described in Delclos et al. [12], except that solid-bottomed polysulfone cages with hardwood chip bedding were used rather than wire bottom cages and a subset of the breeder males (2 cases) were held for remating with a different female that had lost their breeding males. A subset of male breeders was kept, single housed, after mating for use in the hypothesis-driven testes function study. Daily gavage dosing of the dams began on gestation day (GD) 6 (GD 0 = sperm positive day) and continued until the initiation of parturition.

Pups were not dosed on the day of birth (PND 0). Pups were randomly culled to a maximum of five males and five females on PND 1, with tissues from some culls used for hypothesis-driven studies. While a balanced sex ratio after culling was the goal, because the studies required more males than females, the sex distribution of the cull was skewed toward males in later study loads. Litters with fewer than 3 pups/sex and live litters born to dams earlier than GD 20 were excluded from the core study. Litters with at least 6 pups at PND 1 that did not meet the minimum 3 pups/sex criterion were used in the hypothesis-based studies that did not have litter size or sex ratio requirements. Pups with evident malformations (*e.g.*, hydrocephalus) were also excluded from the study. Direct gavage dosing of the pups started on PND 1 after the litter was culled. For pups younger than PND 5, the gavage needle did not enter the esophagus. Before weaning at PND 21, pups were weighed and dosed daily until the scheduled day of removal (PND 15 or PND 21). After weaning, pups were housed two per cage (except for those assigned to the behavior studies, which were housed 2–3 per cage) and either dosed daily until termination (continuous dose arm) or held without further dosing (stop dose arm). Animals in the continuous dosing arm were weighed daily prior to dosing until PND 90 ± 3, after which they were individually weighed weekly. For animals in the stop dose arm, body weights were recorded at weaning and weekly thereafter.

At weaning, up to a maximum of 3 pups/sex/litter were assigned to the core chronic 2 year study. Same-sex littermates were not assigned to the same combination of study dose arm and time of sacrifice. Forty-six to 50 pups/sex/BPA dose group/dose arm and 26 pups/sex/EE_2_ dose group (continuous dose arm only) were assigned to the 2 year study and 20–26 pups/sex/dose group were assigned to the one year interim assessment (Table 1). The remaining pups from those litters with more than 3 same-sex pups were assigned to the hypothesis driven studies at PND 15 (for the thyroid endpoints study) and at weaning.

An additional breeding (“Load 0”, not depicted in Fig. 1) was conducted at the request of one academic researcher for additional evaluation of testes and epididymal sperm. Fourteen dams were assigned to vehicle dose group and 14 dams were assigned to 250,000 μg BPA/kg bw/day and treated as described above. Pups from 10 l per treatment group were then treated daily until PND 90.

## 3. Animal identification

Prior to mating, all *F*_0_ animals were identified by tail tattoo (Animal Identification and Marking Systems, Inc., Hornell, NY) with their unique cage number. *F*_1_ pups were initially numbered on their backs with an indelible marker after culling on PND 1 and were identified quickly on PND 1 after the culling procedure and prior to the start of postnatal weighing and dosing by paw tattoo with a standard 4-paw pattern corresponding to the number indicated on its back. The paw tattoo pattern and dam ID (cage number) provided unique identification for preweaning pups. Retained*F*_1_ pups were marked by tail tattoo with their unique ID (cage number and an additional digit to distinguish cage mates) after weaning on PND 21.

## 4. In-life data collection

Morbidity/mortality checks were performed twice daily and clinical observations were recorded weekly or when a significant clinical observation was noted. Body weights were obtained prior to dosing for dams from GD 6 through parturition and similarly for the pups from PND 1, as described above. Feed consumption was measured weekly from the start of dosing for approximately the next 13 weeks and monthly afterward in the chronic 2 year study, primarily to estimate consumption of background dietary BPA. Water intake was not measured. On the day of birth, the number of pups alive and dead was recorded, but there were no other manipulations of the dam or litter. On PND 1, the number of pups alive and dead, sex ratio, and live litter weight by sex were determined prior to culling. These data will be reported as part of the core chronic 2 year study report.

## 5. Evaluation of the potential for unintentional exposure of CLARITY-BPA animals to BPA

**Fig. 2:**
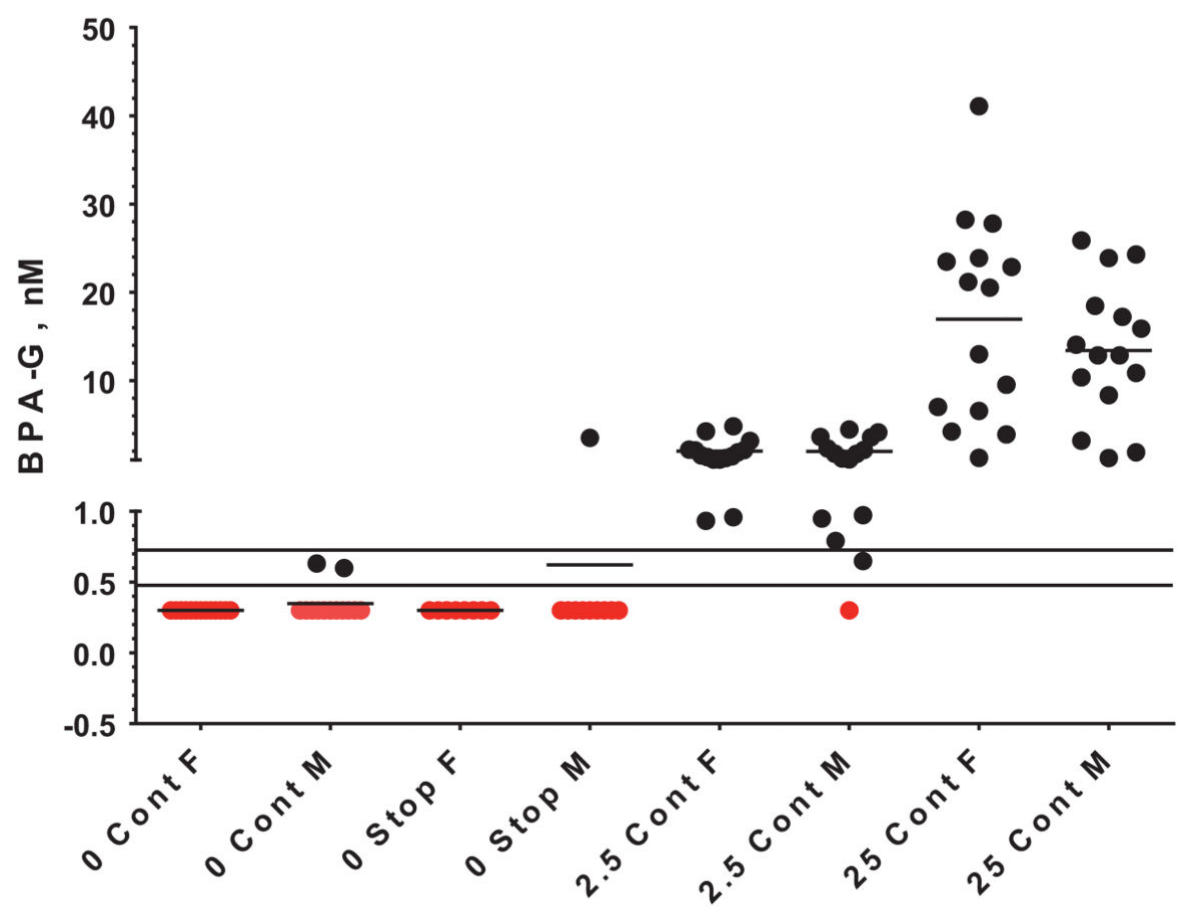
Serum BPA-G measurements in vehicle, 2.5, and 25 μg BPA/kg bw/day animals from the BPA core chronic study. Vehicle (continuous and stop dose arms) and BPA-dosed (continuous arm only) males and females were evaluated. At a single time point within one week of termination at 12 months of age, rats from each of the 5 housing rooms of the core chronic study and representing all 5 study loads were sampled from the tail vein to provide approximately 0.5 ml of blood. The blood sample from animals in the continuous dosing arm was drawn 15–60 min after gavage, approximately *C*_max_ following gavage administration [8]. Rats from the vehicle control stop dose arm were sampled at approximately the same time of the day. Serum samples were analyzed by LC–MS/MS blinded to treatment group. The limit of detection (LOD) was established on each day of analysis and the range is indicated by the two horizontal lines on the graph. Red symbols indicate BPA-G measurements that were <LOD and are indicated as 1/2 LOD. *X*-axis labels, their definitions, and number of animals sampled are as follows: 0 Cont F and 0 Cont M represent continuous dose vehicle control females and males, respectively, *n* = 13/sex; 0 Stop F and 0 Stop M represent stop dose vehicle control females and males, respectively, *n* = 10/sex; 2.5 Cont F and 2.5 Cont M represent continuous dose 2.5 μg BPA/kg bw/day females and males, respectively, *n* = 15/sex; 25 Cont F and 25 Cont M represent continuous dose 25 μg BPA/kg bw/day females and males, respectively, *n* = 15/sex.

After the start of the CLARITY-BPA study, it was found that naïve and vehicle control animals in the NCTR BPA subchronic 90 day study had serum levels of BPA-glucuronide (BPA-G) similar to those produced by the lowest BPA dose of the study (2.5 μg/kg bw/day) [14]. It was hypothesized that there had been an unintentional exposure to BPA resulting from the housing of control animals with the two very high BPA doses (100,000 and 300,000 μg/kg bw/day). Although the highest BPA dose in the CLARITY-BPA study is 25,000 μg BPA/kg bw/day (Loads 1–5), Load 0 animals were treated with 250,000 μg BPA/kg bw/day. Some of these Load 0 animals were housed in the same rooms as the other CLARITY-BPA animals. In the absence of specific internal dosimetry measurements, it was assumed that the CLARITY-BPA study animals housed with the Load 0 high BPA dose (250,000 μg/kg bw/day) might have serum levels of BPA-G above the limit of detection (LOD) of currently available analytical methods, while animals housed in rooms with the high BPA dose of 25,000 μg/kg bw/day would have serum levels of BPA-G below the LOD. Internal dosimetry data collected in CLARITY-BPA study animals support this hypothesis: (1) serum from eight of 10 vehicle-only treated females taken at the PND 90 necropsy after an overnight fast and co-housed in rooms with animals dosed with 250,000 μg BPA/kg bw/day had BPA-G detectable above the LOD1.6 nM ± 0.8 (S.D.)]; (2) serum from 5 vehicle and 5 EE_2_-dosed animals similarly taken at the PND 90 necropsy after a 5–7 h fast, but co-housed in rooms with 25,000 μg BPA/kg bw/day as the highest dose, did not have detectable BPA-G (<LOD); (3) tail vein blood from 5 untreated and unhandled sentinels co-housed in rooms with 25,000 μg BPA/kg bw/day as the highest dose at the time of blood collection did not contain detectable levels of BPA-G; and (4) serum from 1 year core chronic animals co-housed in rooms with 25,000 μg BPA/kg bw/day as the highest dose and collected at approximately *C*_max_ after dosing with vehicle, 2.5, or 25 μg BPA/kg bw/day showed a clear and statistically significant separation of serum levels of BPA-G among the three dose groups (Fig. 2). Of the 46 vehicle serum samples analyzed, only 3 had detectable levels of BPA-G. Two of these samples were within the range of the daily LOD while the third vehicle sample with detectable levels of BPA-G (3.5 nM) was determined to be incorrectly labeled (Fig. 2). We continue to follow this possible contamination issue. The CLARITY-BPA animals housed in the same room as those dosed with 250,000 μg BPA/kg bw/day and the days of co-housing are known, because individual animals can be tracked throughout the study using the NCTR in-life software system; this information will help determine if data from these animals have any influence on the outcome of a particular endpoint in most studies.

### 5.1. Chronic study

The core chronic study was conducted in compliance with the Food and Drug Administration (FDA) Good Laboratory Practice for the conduct of nonclinical laboratory studies (United States Code of Federal Regulations Title 21, Part 58). An interim sacrifice was conducted at 1 year of age (PND 365 ± 20) and the terminal sacrifice was conducted at 2 years of age (PND 730 ± 20). The numbers of animals designated for interim and terminal sacrifice in each dose group are shown in Table 1.

Food, but not water, was removed from animals before scheduled necropsy. Animals were anesthetized with gaseous carbon dioxide and blood was collected from the retro-orbital sinus. Standard hematology and clinical chemistry endpoints (NTP Specifications, 2011 http://ntp.niehs.nih.gov/ntp/test_info/finalntp_reprospecsmay2011 508.pdf), insulin, leptin, cardiac troponins T and I, triiodothyronine (T3), thyroxine (T4), and thyroid-stimulating hormone (TSH) were evaluated in the interim sacrifice. All animals reaching the scheduled terminal date were subjected to a full necropsy. Selected organs were weighed. Tissues not specified for microscopic evaluation were processed to paraffin block and held for potential later evaluation. For tissues specified for evaluation by the study pathologists, all dose groups were evaluated. All gross lesions were processed for histological evaluation. The following organs were examined microscopically: adrenals, aorta (thoracic), bone marrow (femur), brain, right epididymis, heart, kidneys, liver, 5th left mammary gland (inguinal, female and male), ovaries, oviduct, pancreas, parathyroid, pituitary, prostate (dorsolateral and ventral), seminal vesicles with coagulating gland, spleen, right testis, thymus, thyroid, uterus, and vagina. For the dorsolateral prostate, 6 step sections cut at 100 μm intervals were evaluated. Subsets of intermediate sections were collected and stored unstained for potential additional evaluation. The left testis was used for evaluation of testicular spermatid head counts. The left epididymis was used for epididymal sperm counts, morphology, and motility evaluations. Procedures for the 1 and 2 year necropsies were the same, except that the 2 year sacrifice does not include clinical chemistry, hematology, organ weights, or sperm evaluations. The core chronic study data will be analyzed following statistical methods similar to those used in the subchronic 90 day study [12]. Statistical comparisons will be conducted within sex and, for data collected after weaning, within dosing arm (continuous dosing or stop dosing). The five BPA dose groups will be compared to the vehicle control group. Similarly, the two EE_2_ reference estrogen dose groups will be compared to the vehicle control.

## 6. NIEHS-funded hypothesis-driven studies

Integration of molecular, cellular, morphological, and functional endpoints examined by university-based researchers with validated endpoints could contribute additional information in understanding the possible effects of BPA exposure. A major goal of the CLARITY-BPA study was to determine if endpoints reported to be affected by BPA by hypothesis-driven studies are reproducible when assessed under the controlled conditions of a guideline-compliant study. If reproducible, the study could determine if the endpoints previously reported in hypothesis-driven studies at times shorter than two years, might lead to or be correlated with adverse outcomes at later ages, such as 2 years of age.

The university-based researchers are responsible for conducting the laboratory experiments specified in their NIEHS-funded grants and for analyzing and reporting their data. The dose groups, time points, sample size, and endpoints in these studies were determined by the individual university-based researchers. The specimens were provided by NCTR, and included live animals (behavior and erectile dysfunction studies only), tissues, and/or serum. The behavior and erectile dysfunction assays were conducted at the NCTR by staff from the university-based researcher laboratories with assistance from NCTR scientists, while tissue and serum samples were collected by NCTR staff and shipped to the university-based research laboratories for further analyses. Staff members from the university-based research laboratory responsible for the testis function study came to NCTR to process the freshly harvested tissues prior to shipping.

Prior to the start of the study, the university-based researchers provided NCTR with requests for litter-of-origin specifications (*e.g.*, minimum litter size, sex ratio at birth), *in life* data collection (*e.g.*, palpation of animals), and detailed instructions for animal and tissue removal (*e.g.*, assessment of estrous cycle to determine day of sacrifice, fasting requirements) and sample collection and processing (*e.g.*, organ weighed or not, determination of estrous phase at termination, tissue dissection, fixation and freezing, need for sterility or RNase-free conditions, gross lesion processing, serum volume needed).

When possible, the biological samples for a variety of endpoints were collected from the same animals and were shared among university-based researchers, a feature that represents a unique aspect of this project (Table 2). Sharing of multiple tissues per animal allowed not only a significant reduction in animal use, but resulted also in a more robust study design, as it integrated a larger number of often related endpoints collected per animal. On the other hand, a disadvantage of the tissue sharing was that it precluded special treatments of animals that would have been useful for other specific endpoints. The list of university-based researchers and a general description of their endpoints has been published [11]; below further details of the research hypothesis and specific endpoints of each university-based researcher study are described, as well as information on study coordination and on university-based researchers data tracking and decoding.

### 6.1. Reproductive system effects

#### 6.1.1. Male

##### 6.1.1.1. Obstructive voiding disorder (Fred vom Saal, University of Missouri)

The purpose of this project is to determine if developmental exposure to BPA results in periurethral gland enlargement and urethral gland obstruction associated with obstructive voiding disorder in adulthood. The vom Saal group, in collaboration with Dr. William Ricke at the University of Wisconsin-Madison, is examining the periurethral gland structure using computer-assisted reconstruction and morphometric analysis coupled with gene expression collected *via* laser capture microscopy in PND 1 male pups exposed to all doses of BPA and EE_2_. A similar gene expression analysis coupled with histopathology is being performed on 1-year-old animals exposed to all doses of BPA and EE_2_ (continuous dose arm only), to assess the effects of continued exposure beginning during fetal life throughout postnatal life on urethral obstruction at adulthood.

##### 6.1.1.2. Testes function (Kim Boekelheide, Brown University)

This project aims to determine if addition of sophisticated morphological and molecular endpoints to the core chronic study provides increased sensitivity and more specific biomarkers of treatment-related male reproductive effects. The Boekelheide group is integrating data from the core chronic study with additional morphological evaluations, genome-wide microRNA and transcriptomic profiling, and determination of genomic DNA methylation status from both the testis and sperm. Specifically, spermatid head retention in seminiferous tubules, deoxynucleotidyl transferase (TdT)-mediated dUTP nick end labeling (TUNEL) staining in testes to assess apoptotic germ cells in a stage specific manner, altered sperm mRNA and microRNA profiles using whole-genome arrays, and perturbed sperm DNA methylation profiles in CpGenriched regions using reduced representation bisulfite sequencing are being examined [29,30]. Sperm molecular biomarkers of BPA exposure will be developed from PND 90 and 1-year-old animals across all BPA doses and the 250,000 μg BPA/kg bw/day high dose (Load 0) using genome-wide approaches. The high EE_2_ dose group is being analyzed at the 1 year timepoint as well.

##### 6.1.1.3. Erectile dysfunction (Nestor Gonzalez-Cadavid, University of California, Los Angeles)

This project aims to determine if developmental exposure to BPA induces erectile dysfunction and/or affects the underlying penile corpora cavernosal histopathology. At 6 months of age, animals in all continuous doses of BPA are being subjected to cavernosometry and electrical field stimulation of the cavernosal nerve (EFS) to measure erectile function. Global transcriptomic signatures are being obtained from smooth muscle cells cultured from the corpora cavernosa. Serum testosterone and estradiol are being measured at 6 months. Serum, brain, and penile tissue from the 6 month (stop dose arm) and 12 month (continuous dose arm) were collected and stored for possible analysis depending on the results of the 6 month continuous arm samples. The data will be integrated into other aspects of male reproduction assessed by other CLARITY-BPA researchers for a more holistic picture of possible treatment-related effects on the male reproductive system.

#### 6.1.2. Female

##### 6.1.2.1 Ovarian dysfunction and abnormal hormone levels (Jodi Flaws, University of Illinois at Urbana-Champaign)

This project aims to determine the potential of BPA to inhibit follicle growth and induce atresia, leading to low estradiol (E_2_) levels. The Flaws group is examining the effect of BPA on follicle numbers, by assessing follicle growth and atresia, and on E_2_ metabolism, by assessing its synthesis and metabolism in rat ovaries. To accomplish these goals, extensive histological evaluation of the numbers of healthy and dying germ cells, primordial follicles, primary follicles, preantral follicles, and antral follicles at PND 1, PND 21, 6 months, and 1 year are being conducted. All doses of BPA and EE_2_ are being assessed from the continuous and stop dosing arms of the study. Serum estradiol and progesterone levels at PND 21, 6 months, and 1 year are being measured also.

##### 6.1.2.2. Neurobehavioral effects (Heather Patisaul, North Carolina State University; Cheryl Rosenfeld, University of Missouri)

This project evaluates the potential for developmental exposure to BPA to induce subtle transcriptional and epigenetic changes at birth in the hypothalamus and hippocampus and to alter levels of anxiety and activity in juveniles and adults. On PND 1, serum testosterone and estradiol, global DNA methylation and transcriptomics, *via* RNAseq, are being assessed in micropunched sections of the hypothalamus and hippocampus in the 2.5 and 2500 μg/kg bw/day BPA dose groups in both sexes to identify potentially affected genes. Additionally, several behavioral endpoints are being assessed in juvenile and adult animals dosed with 2.5, 25, and 2500 μg BPA/kg bw/day and 0.5 μg EE_2_/kg bw/day through PND 21 (stop dose arm). Juvenile animals are being tested using open field and elevated plus maze (EPM) apparatus, while adult behavioral testing consists of assessment of anxiety and overall activity (EPM, zero maze, and open field) and spatial learning and memory (Barnes maze). Juvenile brains are being examined for volume of sexually dimorphic hypothalamic nuclei using stereology. Adult brains are being assessed to determine if the gene expression and methylation changes that might be observed in the neonates persist into adulthood.

##### 6.1.2.3. Immune dysfunction (Norbert Kaminski, Michigan State University)

This project aims to determine if BPA developmental exposure results in altered immune competence at adulthood in part through changes in leukocyte composition and function through changes in estrogen receptors. The Kaminski group is examining thymus function (weight, cellularity, viability, thymus/bw ratio, and phenotyping for CD3^+^, CD4^+^, CD8^+^and NK cells) at PND 21 and spleen function (weight, cellularity, viability, spleen/bw ratio, and phenotyping for CD3^+^, CD4^+^, CD8^+^, NK cells, NKT cells, CD11b^+^, CD11c^+^, CD127a^+^, MHCII^+^, surface Ig) at PND21, PND 90, 6 months, and 1 year in both sexes and all BPA and EE_2_ doses, in the continuous dosing arm. The Kaminski group is also quantifying potential changes in estrogen receptors (ER) expression (ER alpha and beta, G-protein-coupled ER, and ER-related receptor gamma) and estrogen-sensitive genes known to be involved in leukocyte function in unstimulated naïve splenic immune cell populations and in activated (LPS) leukocytes isolated from BPA treated rats.

##### 6.1.2.4. Metabolic disease (Andrew Greenberg and Beverly Rubin, Tufts University)

This project is examining the potential for developmental BPA exposure to facilitate the development of dysregulated glucose and insulin metabolism at adulthood with potential insulin resistance, type 2 diabetes mellitus, and altered lipid metabolism that could lead to non-alcoholic fatty liver disease (NAFLD). The Greenberg and Rubin groups are examining blood glucose and insulin levels at 1 year and possibly 6 months of age in the continuous and stop dose arms using all BPA and EE_2_ dose groups. Pancreas histology is being assessed also to determine islet cell mass. Additionally, the potential chronology and progression of hepatic lesions at 1 year and possibly 6 months of age and the lipogenic, oxidative, and inflammatory gene expression and lipid accumulation are being assessed in the liver. Those data will be viewed in relation to the serum measurements of glucose and insulin, adipose tissue weights, adipokines and serum fatty acids, triglycerides, and cholesterol levels (measured by other investigators). Sex differences are also being evaluated.

##### 6.1.2.5. Obesity (Nira Ben-Jonathan, University of Cincinnati)

This project aims to determine the potential for chronic BPA exposure to alter adipose tissue functions that result in metabolic dysregulation, regardless of body weight changes. To address the possible effects of BPA on aspects of weight gain and metabolism, the Ben-Jonathan group is examining weight gain and measuring the subcutaneous and visceral fat pad weights in males and females at PND 90, 6 months, and 1 year after continuous BPA or EE_2_ dosing (all doses); body and fat pad weights were collected at necropsy by the NCTR. Fat tissue is being examined immunohistochemically to measure cellularity and macrophage infiltration. Gene expression of 19 adipogenesis or lipid related genes is being measured, including adiponectin, leptin, cytokines, receptors, transcription factors and enzymes related to lipid metabolism. Serum prolactin, leptin, adiponectin, and IL-6 are being evaluated at PND 90, 6 months, and 1 year.

##### 6.1.2.6. Cardiovascular effects (Scott Belcher, University of Cincinnati)

This project is investigating if BPA exposure can result in cardiac pathology. The Belcher group is examining males and females at PND 21, PND 90, 6 months, and 1 year from all BPA and EE_2_ dose groups from the continuous and stop dose arms. General heart tissue structure, left ventricular free wall thickness, and tissue damage and fibrosis are being evaluated. Cardiac hypertrophy is being measured by calculation of myocyte diameter and volume. Fluorescently-labeled cell membrane surfaces will be identified and extracellular surface measurements are being made by image analysis software.

##### 6.1.2.7. Thyroid effects on Brain and Intestine (R. Thomas Zoeller, University of Massachusetts, Amherst)

This project aims to determine the ability of BPA to disrupt thyroid hormone signaling during development. To test whether BPA antagonizes the thyroid hormone receptor (TR), the Zoeller group is evaluating several endpoints of thyroid hormone action in the developing brain (PND 15), including oligodendrocyte development [31], RC3 expression in the hippocampus, thyrotropin-releasing hormone expression in the hypothalamic paraventricular nucleus, and cerebellar histogenesis from all BPA and EE_2_ dose groups. These endpoints are known to be driven by thyroid hormone [32]. The ileum is being evaluated also given that the intestinal epithelium is sensitive to thyroid hormone [33,34]. In addition, serum thyroid hormone levels (total *T*_4_ and TSH) are being evaluated. A parallel experiment with the drug propylthiouracil (PTU), which causes a significant decrease in serum thyroid hormone, is also being conducted to evaluate the sensitivity of the experimental model to perturbations in thyroid hormone levels.

#### 6.1.3. Cancer

##### 6.1.3.1. Mammary (Ana Soto, Tufts University)

This project aims to determine the effects of developmental BPA exposure on the development of pre-neoplastic and neoplastic lesions and on prepubertal (PND21) mammary gland morphology, DNA methylation profiles, and alterations in gene expression. PND 21 data will be used to assess their potential to serve as prognosticators of later pathological outcomes. The Soto group is quantifying preneoplastic and neoplastic lesions in mammary glands at PND 21, PND 90, and 6 months for all BPA and EE_2_ doses in the continuous and stop dose arms. Representative samples of female rats were palpated weekly by NCTR animal care staff from PND 50 to 6 months of age to investigate the development of mammary tumors. The Soto group is examining prepubertal mammary gland morphology by morphometrics at PND 21. Using this tool, 3D image reconstructions of whole mounted PND 21 mammary glands are being generated to provide detailed information on the gland morphology. Additionally, DNA and RNA are being isolated from laser-captured PND 21 mammary epithelial cells or stromal cells for transcriptomal analyses *via* RNAseq and assessment of global patterns of genomic DNA methylation to define if DNA methylation profiles and concomitant alterations of gene expression at PND 21 will be predictors of potential adult pathological outcomes.

##### 6.1.3.2. Uterus (Shuk Mei Ho, University of Cincinnati)

This project is examining the potential for BPA exposure at critical windows of exposure, accompanied by life-long continuous exposure, to increase the risk of uterine cancer in rats. The Ho group is examining all BPA and EE_2_ doses in the continuous dosing arm at 6 months and 1 year in order to determine a potential dose response curve for chronic BPA exposure and development of uterine lesions, uterine atypical hyperplasia, and/or adenocarcinoma, along with evaluations of cell proliferation and apoptosis. If a specific BPA dose results in a high incidence of uterine lesions, that dose will be assessed further using global methylation and transcriptomic analysis to identify novel gene methylation targets driven by BPA and associated with uterine tumor and cancer development. If the expression of certain genes is modified by BPA exposures, those genes will be assessed at PND 21, PND 90, and 6 months to determine their possible relation to uterine tumor formation, as determined in the core chronic study. By comparing the methylome/transcriptome in rat uterus with that in the prostate (see Prins below), it may be possible to evaluate if BPA exposure reprograms genes in a sex and/or tissue specific manner.

##### 6.1.3.3. Prostate (Gail Prins, University of Illinois at Chicago)

This project is determining if developmental BPA exposure alters developmental programing of the prostate epithelial stem cells leading to increased susceptibility to prostate cancer later in life. The Prins group is examining all BPA doses along with the 0.5 μg EE_2_/kg bw/day dose group and the continuous and stop dose arms to determine if there are differences in prostate pathology due to the longer dosing (1 year of age). Some animals were given testosterone + estradiol (T + E) implants at PND 90 because treatment of rats with T + E implants has been reported to induce neoplastic lesions in the prostate [35]. The potential for BPA to alter the incidence of prostatic neoplastic lesions with and without this hormone treatment is being assessed. Prostate pathology, epithelial hyperplasia, and prostatic intraepithelial neoplasia lesions are being examined at 1 year of age. DNA methylation and expression of selected EE_2_ and BPA reprogramed genes in lateral and dorsal prostates are being assessed at 1 year in the stop dose study to identify possible epigenetic reprograming present at that time. To investigate further the mechanisms, stem/progenitor cells are being isolated from prostates from vehicle, BPA (2.5, 25, or 250 μg/kg bw/day) or EE_2_ (0.5 μg/kg bw/day) treated 6 month-old rats (continuous dose arm only) and cultured using a prostasphere assay [36,37]. Differences in prostasphere numbers, size, gene expression, and/or differentiation ability are being evaluated after three passages in the absence and presence of estradiol.

##### 6.1.3.4. Study integration and coordination

The CLARITY-BPA program is overseen by a Steering Committee, which sets consortium policies and resolves conflicts as needed. The Steering Committee is empowered to recommend adjustments to accommodate new knowledge and redirect the scientific focus of the university-based studies as necessary. The Steering Committee includes investigators representing each NIEHS-funded grant, the NCTR Principal Investigator responsible for the core study, a representative from NIEHS's Division of Extramural Research and Training (DERT), a NTP representative responsible for coordinating the project, and the NIEHS-DNTP and FDA-NCTR project officers responsible for administering the interagency agreement that supports the core study. In addition, an External Scientific Panel of three scientists provides overall programmatic guidance and offers advice in the management and technical performance of the research.

The overall consortium policies were compiled into several documents prior to implementation and include the following:

1. Articles of Collaboration. This document describes the specific roles of the Steering Committee and the External Scientific Panel and contains guidelines for conflict resolution. It also describes the details of necropsy and sample collection of the animals for the university-based studies, how samples would be coded, sample shipping, data storage and sharing, and authorship and publication guidelines.
2. Publications Agreement. This document describes the procedures for reviewing of abstracts and manuscripts and the acknowledgments and disclaimers to be included in each publication.
3. Transfer of Data to Chemical Effects in Biological Systems (CEBS) database/CEBS Access Memo/Decoding Standard Procedures. This document delineates the procedure for university-based researcher's data submission to CEBS and the follow-up procedure for sample decoding and lists all expected datasets and estimated timeline for data submission by each university-based researcher. CEBS is a relational database at NIEHS that houses public toxicogenomics data and maintains private repositories before data are made public [38].

## 7. Data tracking and coding/decoding of specimens

Specimens (tissues, serum, and/or live animals) were provided by NCTR to university-based researchers for their analysis without information of the treatment group or dosing arm. In general, specimen identifiers were not grouped in any way; however, in certain instances, additional specimen information was provided to individual university-based researchers, upon presentation of a strong rationale of the need for such data. The requests for additional specimen information were submitted to the consortium's Decoding Team, which is composed of representatives of FDANCTR, FDA-CFSAN, NIEHS-DNTP, and NIEHS-DERT along with one university-based researcher representative, which approved (or denied) the request. Examples of approved additional specimen information include grouping of the specimen identifiers per coded dose group (*e.g.*, need to pool samples or to balance coded dose groups across experiment batches) and listing a subset of coded identifiers to match only a subset of coded dose groups (*e.g.*, only a particular subset of dose groups was to be analyzed). In two instances, partial sample decoding (*i.e.*, grouping of identifiers per coded dose group and identification of the grouped identifiers as vehicle, BPA, or EE_2_) was also granted in order to permit preliminary statistical analysis needed to design follow-up experiments. The partial decoding information was provided to the requesting university-based researcher upon signing of a confidentiality agreement.

Serum and tissues collected from a given animal were each labeled with a different set of identifiers; in addition, all tissues collected from a given animal were labeled with the same identifier. This was necessary to minimize the chance of specimen mislabeling, due to the already complex logistics during necropsy. A measure implemented in the 6 and 12 month time points, which alleviated considerably the burden of shared decoding, was to relabel the fixed tissues with unique identifiers prior to shipping to the university-based researcher laboratories. Frozen specimens were not relabeled to avoid the potential for sample thawing and resulting degradation.

Coded raw data, along with an explanation for any missing data, were submitted by the university-based researchers to the CEBS Administrator. As each data set was received, the CEBS Administrator or designee performed a completeness check against NCTR's coded identifier list. Missing data were documented in writing and reported to the university-based researcher for resolution by the Decoding Team. Once completed and upon approval by the Decoding Team, the raw data sets were archived in a read-only repository in CEBS. Once all expected data were submitted for a given animal set, the data were decoded. For animal sets shared by multiple university-based researchers, the data were decoded upon archiving of all expected data from the multiple university-based researchers; approval was required by the Decoding Team and all university-based researchers before the decoding process began. Decoding was performed by the CEBS Administrator using the decoding information provided by NCTR that linked, where applicable, the specimen identifiers with the treatment group, dosing arm, litter of origin, sex, windows of overlap with Load 0, estrous phase on the day of sacrifice, and body and organ weights. Accuracy was verified by the CEBS Administrator by performing quality assurance for data integrity and verification of decoding information. When the decoding process was complete, approval for release was granted by the Decoding Team. At this point university-based researchers were provided access to the verified decoded data in CEBS. For quality assurance purposes, all coded and decoded data, correspondence, and the decoded identifier lists remain archived in CEBS.

## 8. Discussion

CLARITY-BPA is a large-scale, multi-year consortium with a goal of bringing together the strengths of a guideline-compliant study and hypothesis-driven studies. It makes additional use of animals produced in guideline-compliant reproductive studies, since these studies typically analyze one pup per sex per litter/time point. The use of the otherwise surplus littermates to generate additional cellular, molecular, and disease-focused endpoints in the hypothesis-driven experiments by university-based researchers will result in a more powerful study design, with the potential to integrate multiple, related endpoints because datasets are collected using a common animal model, diet, and dosing regimen. The inlife phase of the university-based research studies was completed in January 2014, and the in-life phase of the core chronic study was completed in January 2015. Many of the expected university-based researcher datasets have been archived in CEBS and decoded, and reporting of the data either in scientific meetings and through peer-reviewed articles is ongoing. Data from the core chronic study is expected to be publicly available by 2018. In addition, positive and negative raw data from all studies are intended to be archived in CEBS, along with information for each study animal on the load and litter of origin, estrous cycle phase at sacrifice (for cycling females), and windows of overlap with Load 0 (to enable identification of potential periods of unintentional exposure to environmental BPA) will be made publically available at study completion to offer the possibility for others to conduct independent analyses.

Although the study is still ongoing, several lessons have been learned thus far. Effective and open communication among consortium participants during all phases of the study, from design to data collection to final data analyses or presentation, has been critical and will continue through the public presentation and the publication of data. Representatives from all CLARITY-BPA stakeholders, including NIEHS-DERT, NIEHS-NTP, FDA-NCTR, FDA-CFSAN, and a university-based researcher representative, met by teleconference weekly for the first year of the study and approximately biweekly for the remainder of the study to follow study progress and address any unforeseen problems. In addition, webinars with all CLARITY-BPA participants, including the External Scientific Panel, are held periodically to report the progress of chronic and other studies and to discuss consortium-wide issues. Given the large number of participants in the consortium and the various timelines for data collection amongst the hypothesis-driven studies, it is essential to have specific rules and guidelines defined at the onset of the program. For CLARITY-BPA, we developed a set of articles of collaboration, confidentiality statements, publication agreements, memos describing transfer of data to the CEBS database, and an SOP for decoding of all data, which have been critical to the functioning of the program. This consortium research model may be useful for investigating BPA by shedding light on doses, endpoints, and methods that may enhance the utility of traditional toxicology study designs for investigating additional chemicals. It is also possible that future collaborative programs based on this model will increase the impact of basic research and to chemical risk assessments.

## Figures and Tables

**Table 1:** Number of animals/treatment group assigned to the interim (1 year) and terminal (2 year) sacrifices of the core chronic BPA toxicity study.^a^

Group	Arm (Continuous/Stop)	1 Year		2 Year	
	
		Male	Female	Male	Female
Vehicle	Continuous	22	23	50	50
	Stop	20	20	50	50 (49)^b^
2.5 BPA	Continuous	22	22	48	48
	Stop	20	22	48	50
25 BPA	Continuous	20	22	48	46
	Stop	20	20	48	48
250 BPA	Continuous	24	24	50	50
	Stop	20	22	50	50
2500 BPA	Continuous	20	20	50	50
	Stop	20	20	50	50
25,000 BPA	Continuous	22	24	46	46
	Stop	22	22	46	46
0.05 EE_2_	Continuous	26	26	26	26
0.50 EE_2_	Continuous	26	26	26	26

a Animals were allocated to the interim or terminal sacrifice of the continuous or stop dose arms of the study at weaning. There were no same sex litter mates in any treatment group. The original protocol indicated 26 animals/sex/treatment group for the interim sacrifice and 50 animals/sex/BPA treatment group (26 for EE_2_ groups) for the terminal sacrifice. A shortfall in the number of pups available for the specialty studies resulted in a change such that 20–26 animals/sex/treatment being allocated to the interim sacrifice and any extra pups were allocated to specialty studies.

b In this group, one animal assigned was found to have been incorrectly sexed and was discarded on the day of allocation.

**Table 2:** Summary of functional assays and specimens collected per animal set in the hypothesis-driven studies.

Animal set by age at sacrifice	Functional assays and specimens collected at necropsy	Other information
PND 1	Males: serum, brain, UGS with bladder Females: serum, brain, ovary	Animals originated from litters with a minimum of 9 pups born and a balanced sex ratio at birth (no more than two extra pups of either sex, if possible)
PND 15	Males and females: serum, brain, heart, ileum, liver, pituitary	Additional animals were treated with vehicle or PTU
PND 21	Males: serum, heart, spleen, thymus Females: serum, heart, mammary gland, ovary, spleen, thymus, uterus	
PND 21	Females: serum, mammary gland	
PND 90	Males: serum, eye, fat pads, heart, prostate, spleen Females: serum, eye, fat pads, heart, mammary gland, ovary, spleen, uterus	Animals were fasted 5–7 h prior to sacrifice and cycling females were scheduled to be sacrificed at estrus
PND 90	Males: testes, epididymal sperm	Additional animals were treated with vehicle or 250,000 μg BPA/kg bw/day
6 months	Males: serum, brain, fat pads, heart, liver, pancreas, penis, prostate, spleen Females: serum, brain, fat pads, heart, liver, mammary gland, ovary, pancreas, spleen, uterus	Animals were fasted 5–7 h prior to sacrifice and cycling females were scheduled to be sacrificed at estrus
6 months	Males: erectile function assessment, serum, brain, penis	
1 year	Males: serum, fat pads, heart, liver, pancreas, prostate, spleen Females: serum, fat pads, heart, liver, ovary, pancreas, spleen, uterus	Animals were fasted 5–7 h prior to sacrifice and cycling females were scheduled to be sacrificed at estrus.
1 year	Males: prostate	Animals were implanted with T + E at PND 90
1 year	Males: testes, epididymal sperm	
1 year	Males: urinary tract	Animals originated from litters with a minimum of nine pups born and a balanced sex ratio at birth (no more than two extra pups of either sex, if possible)
Juvenile and adult	Males and females: behavior assessment, serum, brain	Animals originated from litters with a minimum of 9 pups born and a balanced sex ratio at birth (no more than two extra pups of either sex, if possible)

## Abbreviations

BPA: bisphenol A
CLARITY-BPA: Consortium Linking Academic and Regulatory Insights on BPA Toxicity
NIEHS: National Institute of Environmental Health Sciences
FDA: United States Food and Drug Administration
NTP: National Toxicology Program
